# Integrated systems toxicology identifies TCDD-responsive targets linked to immune dysregulation and treatment response in psoriasis

**DOI:** 10.3389/fmed.2026.1816748

**Published:** 2026-05-29

**Authors:** Xuan Zhang, Yankun Zhang, Yanling He

**Affiliations:** Department of Dermatology, Beijing Chao-Yang Hospital, Capital Medical University, Beijing, China

**Keywords:** psoriasis, 2, 3, 7, 8-tetrachlorodibenzo-p-dioxin, network toxicology, immune dysregulation, treatment response

## Abstract

**Background:**

Psoriasis is a chronic immune-mediated inflammatory skin disease driven by dysregulation of the IL-23/IL-17 axis and influenced by genetic and environmental factors. The role and molecular mechanisms of the environmental pollutant dibenzo-p-dioxins, particularly 2,3,7,8-tetrachlorodibenzo-p-dioxin (TCDD), in psoriasis remain unclear.

**Methods:**

An integrative strategy combining network toxicology, machine learning, bioinformatics analysis, molecular simulation, and *in vitro* keratinocyte qRT–PCR validation was employed to systematically investigate the potential toxicity and molecular mechanisms of TCDD in psoriasis.

**Results:**

Eighty-seven overlapping genes were identified between TCDD-related targets and psoriasis-associated genes and were mainly enriched in IL- 17-, chemokine-, MAPK/ ERK-, and GPCR-related signaling pathways. Machine learning identified five core target genes—LCK, MMP9, CXCR2, PTAFR, and CCNB1—which were significantly upregulated in psoriatic lesions, showed strong diagnostic performance within the analyzed datasets, and were associated with local immune infiltration patterns. Structural analyses supported potential interactions between TCDD and these core targets, with CXCR2 showing the most favorable predicted docking score. A 24-h keratinocyte TCDD-response signature showed significant concordance with psoriatic lesional transcriptomes, and keratinocyte qRT–PCR validation showed increased expression of LCK, MMP9, CXCR2, and PTAFR, whereas CCNB1 showed only a modest change. In biologic-therapy cohorts, core genes were downregulated after 12 weeks, and higher baseline MMP9 was associated with poorer clinical improvement and may have potential relevance to treatment response.

**Conclusion:**

Our integrative analyses identify TCDD-associated genes and pathways potentially involved in psoriasis immune dysregulation. Structural modeling supports the *in silico* plausibility of TCDD engaging CXCR2, while transcriptomic concordance and keratinocyte qRT–PCR findings support dysregulation of several core genes in epidermal cells. Baseline MMP9 was associated with week-12 treatment response in biologic-therapy cohorts. Nevertheless, the link between environmental TCDD exposure and psoriasis remains inferential because the study relied mainly on *in silico* analyses without individual-level exposure data; therefore, further validation in well-designed cohort studies is needed.

## 1 Introduction

Psoriasis is a chronic immune-mediated inflammatory skin disease characterized by excessive keratinocyte activation and persistent immune dysregulation, with the IL-23/IL-17 axis playing a central pathogenic role (1, 2). In psoriatic lesions, keratinocytes function as active amplifiers of inflammation by producing cytokines, chemokines, and antimicrobial mediators in response to inflammatory cues, thereby promoting the recruitment and activation of dendritic cells, neutrophils, and pathogenic T-cell subsets and sustaining a feed-forward inflammatory loop (3). In parallel, dysregulated TNF-, IL-23/IL- 17-, chemokine-, and MAPK/ERK-related signaling pathways contribute to epidermal hyperplasia, leukocyte trafficking, and chronic tissue inflammation (4). Consistent with the central importance of these pathways, biologic agents targeting TNF, IL-23, and IL-17 have substantially improved outcomes in moderate-to-severe psoriasis (5, 6). However, variability in treatment response suggests that psoriasis is biologically heterogeneous and may be influenced by additional upstream modulators beyond canonical cytokine circuits.

Emerging evidence suggests that environmental pollutants may contribute to psoriasis pathogenesis by inducing oxidative stress, disrupting epidermal barrier integrity, and promoting immune dysregulation (7). Among these pollutants, dibenzo-p-dioxins have attracted particular attention because of their persistence, bioaccumulation, and toxicological relevance (8). Structurally, 2,3,7,8-tetrachlorodibenzo-p-dioxin (TCDD) is a chlorinated congener derived from the dibenzo-p-dioxin scaffold and represents the most extensively studied and toxic prototype in this group (9). TCDD is generated as an unintended byproduct of chlorine-related industrial processes, waste incineration, open burning of chlorinated materials, and high-temperature metallurgical activities (10). Because of its strong hydrophobicity and affinity for particles, TCDD is widely distributed in environmental matrices, particularly in sediments and dust. In addition to direct environmental contact, dietary intake represents a major route of human exposure, as TCDD readily bioaccumulates in animal-derived foods and contributes to chronic low-level exposure in the general population (8, 10).

Accumulating evidence indicates that TCDD can perturb epidermal homeostasis, inflammatory signaling, and tissue remodeling (11). Classic toxicological studies have shown that TCDD induces marked cutaneous abnormalities, including altered keratinocyte differentiation, barrier dysfunction, and inflammatory responses (12). Although some of these effects have been linked to xenobiotic-responsive pathways, the downstream molecular programs most relevant to chronic inflammatory skin diseases remain incompletely understood. This issue is particularly relevant to psoriasis, in which environmentally responsive signals may converge with pathogenic epidermal and inflammatory circuits (13). Nevertheless, the molecular basis by which dibenzo-p-dioxins, and TCDD in particular, may influence psoriasis remains poorly defined.

In this study, we integrated network toxicology, transcriptomic analysis, machine learning, and *in vitro* validation to characterize TCDD-associated molecular programs in psoriasis. By combining TCDD-related targets with psoriasis-associated genes and applying differential expression and feature-selection analyses, we identified five core genes potentially linking TCDD exposure to psoriatic immune dysregulation. We further assessed their immune relevance, diagnostic value, structural plausibility, and associations with biologic treatment response, and performed keratinocyte-based *in vitro* validation of core-gene regulation. Collectively, these findings provide a systems-level view of TCDD-associated molecular programs in psoriasis and highlight candidate genes and pathways linking environmental toxicant exposure to immune dysregulation, keratinocyte-driven inflammatory remodeling, and therapeutic responsiveness.

## 2 Materials and methods

### 2.1 Target genes collection and integration

Potential targets of TCDD were obtained by retrieving its chemical structure and corresponding simplified molecular-input line-entry system (SMILES) from the PubChem database, followed by target prediction using the ChEMBL, STITCH (confidence score ≥ 0.4), and SwissTargetPrediction databases (accessed on 30 December 2025), with the species restricted to Homo sapiens. In parallel, psoriasis-related genes were collected from the Online Mendelian Inheritance in Man (OMIM) and GeneCards databases (accessed on 30 December 2025). For both datasets, candidate targets from different sources were merged, and duplicate genes were removed to generate non-redundant sets of TCDD-related targets and psoriasis-associated genes, which were subsequently used for downstream analyses.

### 2.2 Target genes functional pathway analysis

To elucidate the biological functions and signaling pathways of TCDD-related target genes in psoriasis, we used the clusterProfiler package in R to perform Gene Ontology (GO) functional annotation and Kyoto Encyclopedia of Genes and Genomes (KEGG) pathway enrichment analysis. The resulting KEGG enrichment data were further processed to construct a chord diagram with Charticulator, enabling visualization of gene–pathway interaction patterns at the network level.

### 2.3 Data processing

Public transcriptomic datasets were retrieved from the Gene Expression Omnibus (GEO) and processed in a unified pipeline. For psoriasis discovery and diagnostic evaluation, GSE13355 and GSE14905 were downloaded and merged after harmonizing gene identifiers. For therapeutic-response analyses, transcriptomic and clinical data from GSE117468 and GSE117239 were integrated after batch-effect correction and normalization. Batch effects attributable to dataset origin were adjusted using removeBatchEffect in the limma package, and the effectiveness of batch correction was evaluated by principal component analysis (PCA) (14). For single-cell transcriptomic analysis, publicly available single-cell RNA-seq data from human skin tissue (GSE162183) were re-analyzed to define the cellular distribution of the five prioritized genes. For keratinocyte TCDD perturbation, GSE226045 was used to construct a 24-h TCDD-response signature. Samples were annotated into TCDD 24 h and time-matched control 24 h groups based on GEO metadata. Supplementary Table 1 presents detailed dataset information, including the microarray platform, sample groups, and numbers.

### 2.4 Identification of DEGs

Differential expression was performed using limma. Gene-wise linear models were fitted with lmFit, followed by empirical Bayes moderation using eBayes (trend = TRUE). For the merged psoriasis cohort (GSE13355 + GSE14905), DEGs were defined by comparing lesional vs. normal skin, applying Benjamini–Hochberg correction and thresholds of FDR < 0.05 and | log2FC| > 0.58. For GSE226045, differential expression between TCDD (24 h) and control (24 h) was assessed using the same framework; genes were ranked by moderated t-statistics to define top 500/1,000 upregulated and top 500/1,000 downregulated TCDD signatures for subsequent preranked GSEA against the psoriasis ranked transcriptome.

### 2.5 Identifying core targets using machine learning

Two complementary machine learning strategies—Least Absolute Shrinkage and Selection Operator (LASSO) regression and Support Vector Machine–Recursive Feature Elimination (SVM-RFE)—were employed to screen for robust core genes associated with TCDD-associated psoriasis (15, 16). LASSO was implemented using the glmnet R package with an L1 penalty (alpha = 1) under a binomial model, with standardized predictors; the optimal regularization parameter (λ) was selected by 10-fold cross-validation (nfolds = 10). Genes with non-zero coefficients at the optimal lambda value (lambda.min) were retained as LASSO-selected features. Simultaneously, SVM-RFE analysis was implemented using the caret and e1071 R packages with a radial basis function kernel (svmRadial) under a 10-fold cross-validation framework to evaluate feature subsets of varying sizes. The optimal feature set was determined based on cross-validated classification performance. Finally, core genes were defined as the intersection of features selected by both the LASSO and SVM-RFE algorithms, representing robust candidate targets associated with TCDD-related psoriasis.

### 2.6 Differential expression of core genes and assessment of diagnostic accuracy

To characterize the expression patterns and diagnostic relevance of the five prioritized core genes, their expression values were extracted from the batch-corrected merged psoriasis cohort (GSE13355 + GSE14905) described above. Group differences between lesional and normal skin were visualized using violin/box plots. Diagnostic performance of each core gene was evaluated by receiver operating characteristic (ROC) analysis using the pROC package in R, and the area under the curve (AUC) was calculated to quantify discriminatory ability.

### 2.7 Immune infiltration analysis

Immune cell infiltration was estimated in the psoriasis cohort using a reference-based deconvolution approach with a modified CIBERSORT algorithm and the LM22 signature matrix, which represents 22 immune cell subsets (17). The normalized expression matrix described above was used as input for deconvolution. Spearman rank correlation analysis was performed to assess associations between the expression levels of the five core genes and the estimated immune cell infiltration scores.

### 2.8 Single-cell transcriptomic analysis of psoriatic skin lesions

Publicly available single-cell RNA-seq data from human skin tissue (GSE162183) were re-analyzed to characterize the cellular distribution of the five prioritized genes. Raw count matrices were processed in Seurat v5 following a standard workflow, with reference to a previously reported psoriasis single-cell analysis (18). Cells with fewer than 200 detected genes, more than 7,000 detected genes, or mitochondrial transcript fractions greater than 20% were excluded. After normalization, 2,000 highly variable genes were identified for downstream analysis, followed by scaling, principal component analysis (PCA), reciprocal PCA-based sample integration, UMAP dimensional reduction, and graph-based clustering. Cell types were annotated according to canonical marker genes for keratinocytes, dendritic cells, macrophage/myeloid cells, lymphoid cells, and melanocytes. The expression of LCK, MMP9, CXCR2, PTAFR, and CCNB1 was then assessed across annotated cell populations using UMAP feature plots and dot plots.

### 2.9 Molecular docking

The three-dimensional crystal structures of core target proteins were obtained from the Protein Data Bank (PDB). The molecular structure of TCDD was retrieved from the PubChem database. Protein structures were prepared by removing water molecules and adding polar hydrogens, followed by docking using AutoDockTools for preparation and AutoDock Vina for scoring and pose generation (19, 20). The binding conformations with the most favorable binding energies were visualized and analyzed using PyMOL and Discovery Studio to examine key protein–ligand interactions.

### 2.10 Molecular dynamics simulation

MD simulations of the TCDD–CXCR2 complex were performed using GROMACS (21). Ligand parameters were generated using AmberTools 22 and the General Amber Force Field (GAFF) (22). Hydrogen atoms were added, and the restrained electrostatic potential (RESP) charge was calculated using Gaussian 16W (23). The resulting ligand parameters were integrated into the system topology of the MD simulations.

The protein–ligand complex was solvated in an explicit TIP3P water model, and system electroneutrality was achieved by the addition of Na^+^ counterions (24). The CHARMM36 force field was applied to the protein (25). Energy minimization was carried out using the steepest descent algorithm, followed by equilibration under constant volume (NVT) and constant pressure (NPT) conditions to stabilize temperature and pressure. Subsequently, production MD simulations were performed under NPT conditions at 300 K and 1 bar for a total duration of 100 ns.

### 2.11 Transcriptomic signature concordance analysis

Transcriptomic concordance between the keratinocyte TCDD signature and the psoriasis lesional transcriptome was evaluated by preranked GSEA. The TCDD-24 h UP/DOWN signatures derived from GSE226045 and the psoriasis lesional preranked list derived from the integrated GSE13355 + GSE14905 analysis (both generated as described above) were used as inputs. Preranked GSEA was performed using the fgsea package in R with permutation-based significance testing, and enrichment was summarized by enrichment score (ES), normalized enrichment score (NES), and false discovery rate (FDR). Statistical significance was defined as FDR < 0.05.

### 2.12 *In vitro* keratinocyte dioxin exposure and qRT–PCR validation of core genes

The human keratinocyte cell line HaCaT was cultured in Dulbecco’s modified Eagle’s medium (DMEM) supplemented with 10% fetal bovine serum and 1% penicillin–streptomycin at 37°C in a humidified incubator with 5% CO2. Cells were treated with dioxin (dibenzo-p-dioxin, 10 nM) or vehicle control (0.1% DMSO) for 24 h. Total RNA was extracted and reverse-transcribed to cDNA, and expression of the five core genes was quantified by qRT–PCR. Relative expression was calculated using the 2^−ΔΔCt method and normalized to GAPDH. Differences between the treated and vehicle groups were assessed using a two-tailed unpaired *t*-test, with *P* < 0.05 considered statistically significant.

### 2.13 Assessment of TCDD-related core genes in relation to therapeutic response

Therapeutic-response analyses were conducted using two psoriasis biologic-treatment cohorts, GSE117468 and GSE117239, after data integration as described above. Paired lesional samples collected at baseline and week 12 were used to assess treatment-associated expression changes of the five core genes. Clinical response was evaluated using ΔPASI, defined as PASI at baseline minus PASI at week 12, and patients achieving PASI75 at week 12 were classified as responders. Associations between treatment-induced gene expression changes (ΔExpr) and ΔPASI were evaluated using Spearman correlation, and baseline expression levels were further tested for association with ΔPASI, including treatment-stratified analyses where applicable. Multiple testing was controlled using the Benjamini–Hochberg procedure, and adjusted P (FDR) < 0.05 was considered statistically significant.

## 3 Results

### 3.1 Network toxicology analysis of potential targets of TCDD-related psoriasis

**FIGURE 1:**
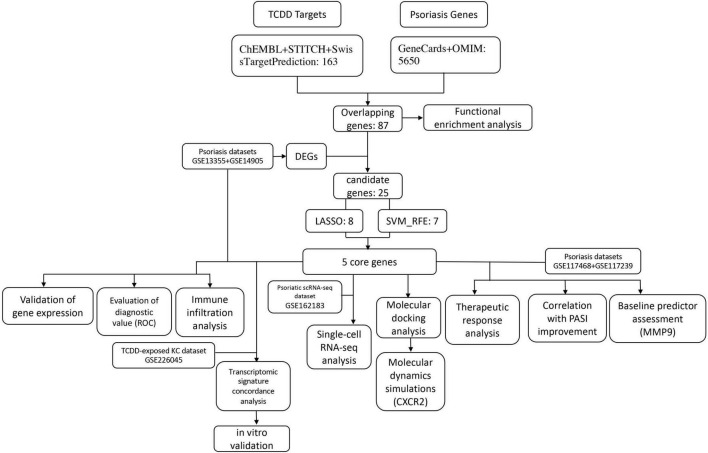
Flow chart of data collection and analysis.

**FIGURE 2:**
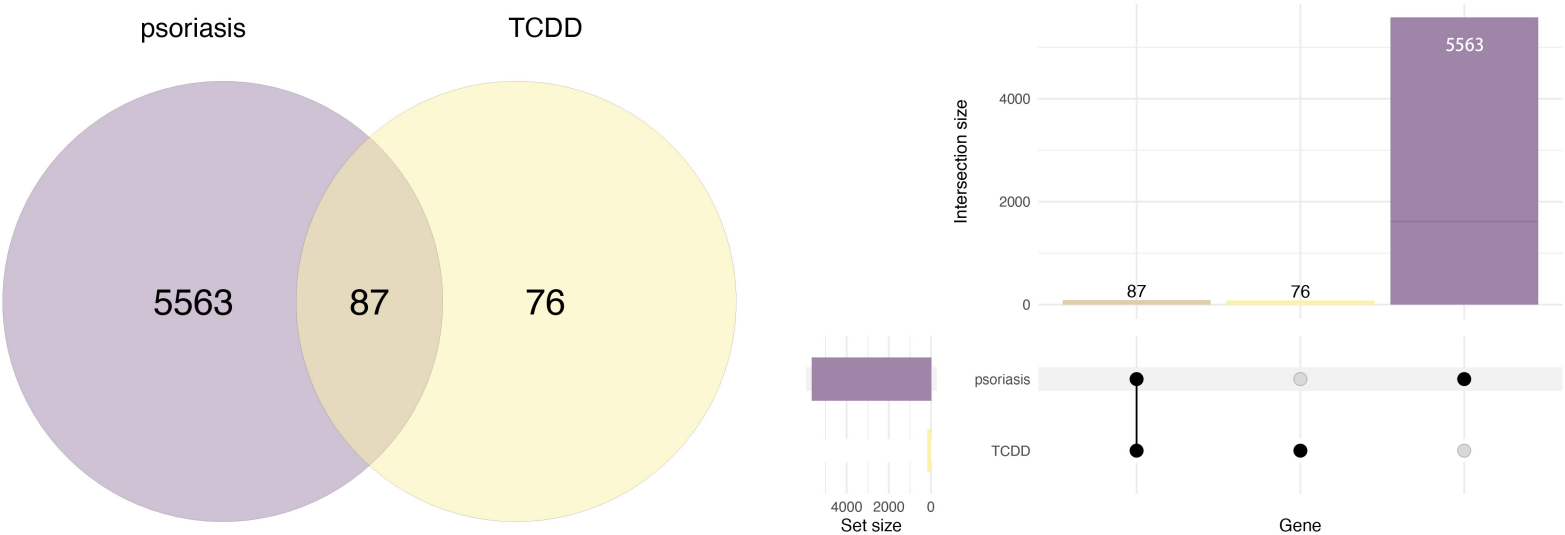
Identification of overlapping targets between TCDD and psoriasis. Venn diagram (left) and UpSet plot (right) showing the overlap between predicted TCDD-related targets and psoriasis-associated genes. A total of 87 overlapping genes were identified.

Figure 1 summarizes the overall workflow of our network toxicology analysis. Network toxicology is an advanced method for predicting the mechanisms and potential targets of environmental toxicants. After deleting duplicates, we identified 163 predicted targets of TCDD by querying the ChEMBL, SwissTargetPrediction, and STITCH databases. At the same time, through an in-depth analysis of GeneCards and OMIM, a total of 5,650 psoriasis-associated targets were identified following data integration and deduplication. Integrating these two gene sets yielded 87 overlapping genes identified as TCDD-associated targets in psoriasis (Figure 2). A complete list of these targets is provided in Supplementary material.

### 3.2 Functional enrichment analysis of TCDD-associated psoriasis targets

**FIGURE 3:**
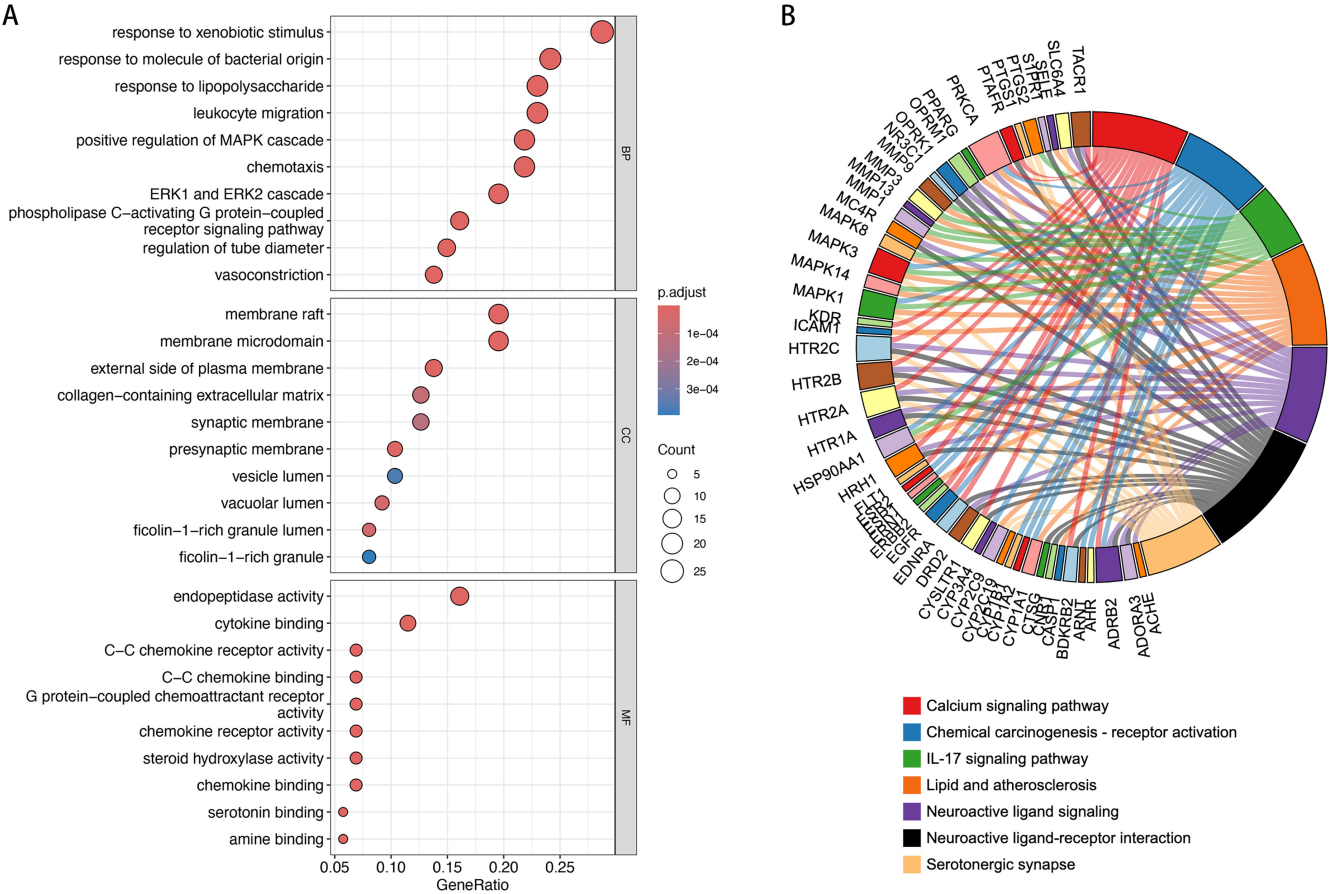
Functional enrichment analysis of TCDD–psoriasis overlapping targets. **(A)** GO enrichment analysis of overlapping targets, including BP, CC, and MF. **(B)** KEGG pathway enrichment analysis visualized by a chord diagram.

To investigate the potential mechanisms underlying TCDD- associated psoriasis, we performed enrichment analyses on the 87 overlapping target genes. Then, the top 10 GO terms with the lowest false discovery rate (FDR) across Biological Process (BP), Cellular Component (CC), and Molecular Function (MF) were visualized (Figure 3A). In the BP category, the TCDD–psoriasis overlapping targets were predominantly enriched in “response to xenobiotic stimulus,” “leukocyte migration” and positive regulation of MAPK cascade and ERK1/ERK2 signaling. In the CC category, enriched terms including membrane raft, membrane microdomain, and external side of plasma membrane highlight that these targets are predominantly localized to membrane structures associated with signal transduction. In the MF category, significant enrichment was observed in chemokine binding, chemokine receptor activity, and G protein–coupled chemoattractant receptor activity.

KEGG pathway analysis identified the top 30 signaling pathways using the clusterProfiler R package, displayed as bubble charts (Supplementary Figure S1). We found that the overlapping targets were mainly involved in neuroactive signaling, immune inflammation, signal transduction, and xenobiotic-responsive processes, including the neuroactive ligand–receptor interaction, IL-17 signaling pathway and calcium signaling pathway (Figure 3B). These pathways were closely associated with immune-related signaling, chemotactic regulation, and cellular stress responses, with key genes such as MAPK1, MAPK3, EGFR, ERBB2, CCR5, and CXCR2 enriched across multiple pathways.

### 3.3 Identification of key targets using machine learning

To further prioritize psoriasis-relevant targets for downstream feature selection, we intersected the TCDD–psoriasis overlapping targets with the differentially expressed genes (DEGs) identified from psoriasis lesional transcriptomic data. The resulting intersection gene set was defined as candidate genes and used as the input feature pool for subsequent machine learning–based target selection.

**FIGURE 4:**
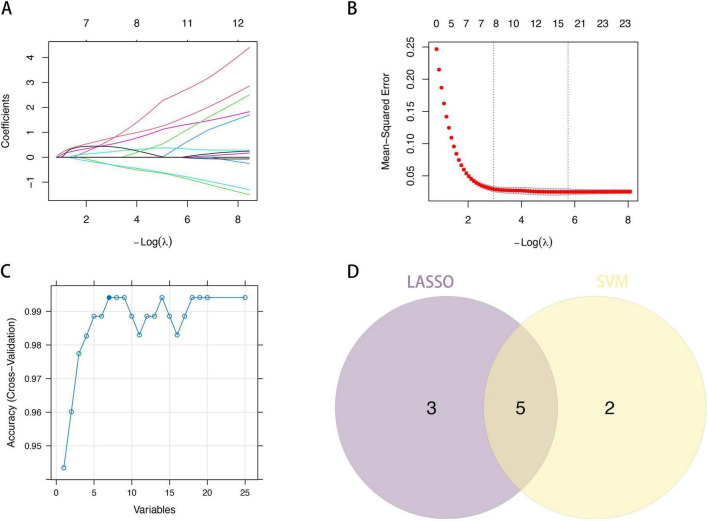
Identification of core genes associated with TCDD- related psoriasis using machine learning. **(A)** LASSO coefficient profiles of candidate genes across different penalty parameters (log λ). **(B)** Ten-fold cross-validation curve of the LASSO model, with 8 genes selected at the optimal λ value. **(C)** Feature selection using the SVM-RFE algorithm, identifying 7 candidate genes. **(D)** Venn diagram showing the overlap between genes selected by LASSO and SVM-RFE. The intersecting genes were defined as core genes.

LASSO and SVM-based machine learning algorithms were subsequently applied to identify key target genes from this candidate gene set. Using LASSO regression with ten-fold cross-validation, a total of 8 genes with non-zero coefficients were identified as candidate core targets (Figures 4A,B). In parallel, SVM-RFE analysis further refined the feature set and identified 7 genes with optimal discriminatory performance (Figure 4C). Integration of the results from both algorithms using a Venn diagram revealed five overlapping genes, which were defined as core candidate targets associated with TCDD-related psoriasis: LCK, MMP9, CXCR2, PTAFR, and CCNB1 (Figure 4D).

### 3.4 Validation of core genes expression and evaluation of diagnostic performance in psoriasis

**FIGURE 5:**
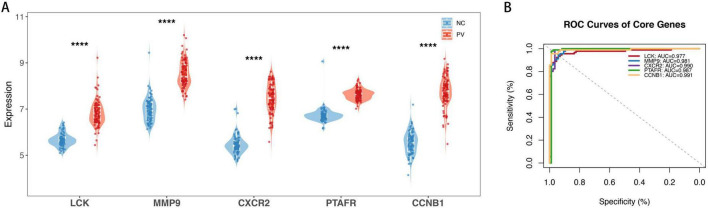
Expression patterns and diagnostic performance of core genes in psoriasis. **(A)** Violin plots showing the expression levels of LCK, MMP9, CXCR2, PTAFR, and CCNB1 in normal skin (NC) and psoriatic lesions (PV) from the merged GSE13355 and GSE14905 datasets. **(B)** ROC curves evaluating the diagnostic performance of the five core genes. ****P < 0.0001.

To validate the expression patterns of the identified core targets in psoriasis, transcriptomic data from GSE13355 and GSE14905 were integrated after batch-effect correction and normalization, as confirmed by principal component analysis (Supplementary Figure S2). Comparative expression analysis showed that LCK, MMP9, CXCR2, PTAFR, and CCNB1 were significantly upregulated in psoriatic lesion samples compared to normal skin (Figure 5A). To further assess the diagnostic potential of these genes, we performed ROC curve analysis. The areas under the curve (AUC) values for LCK, MMP9, CXCR2, PTAFR, and CCNB1 were 0.977, 0.981, 0.990, 0.987, and 0.991, respectively (Figure 5B), suggesting strong discriminative performance in the analyzed datasets. Because these diagnostic analyses were performed within the same cohort used for feature selection, the observed performance should be interpreted as internal evaluation within the analyzed datasets. In summary, these results indicate that the identified core genes are closely associated with psoriasis and represent candidate markers for further diagnostic and mechanistic investigation.

### 3.5 The correlation analysis between core genes and immune cells in psoriasis

**FIGURE 6:**
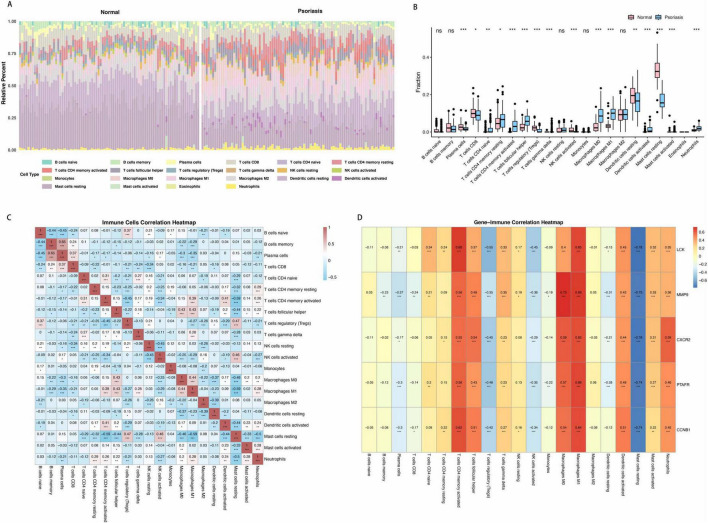
Immune cell infiltration characteristics in psoriatic lesions. **(A,B)** Relative proportions of 22 immune cell types in psoriatic lesions and normal skin estimated by CIBERSORT and visualized using stacked bar plots and box plots. **(C)** Correlation heatmap showing relationships among immune cell types in psoriatic lesions. **(D)** Heatmap showing correlations between core genes and immune cell infiltration levels. *P < 0.05, **P < 0.01, ***P < 0.001.

We characterized the immune cell infiltration profiles in normal skin and psoriatic lesions using a CIBERSORT-based deconvolution method. Estimated immune cell proportions differed between the two groups, suggesting altered immune infiltration patterns in psoriatic lesions (Figures 6A,B). Furthermore, correlation analysis among immune cell subsets revealed multiple associations between different estimated immune cell populations (Figure 6C). Activated dendritic cells showed positive correlations with activated CD4^+^ memory T cells and neutrophils, whereas regulatory T cells were negatively correlated with several effector immune cell populations, including activated CD4^+^ memory T cells and macrophage subsets. Moreover, the differing correlation patterns among macrophage subtypes and T-cell subsets were consistent with the heterogeneity of the immune microenvironment. Overall, these findings suggest that psoriasis lesions exhibit complex immune infiltration patterns involving both innate and adaptive immune cell populations.

Subsequently, we analyzed the relationships between the five core targets and immune cell infiltration (Figure 6D). All five core genes showed positive correlations with the estimated infiltration scores of activated CD4^+^ memory T cells, T follicular helper cells, γδ T cells, macrophage subsets, neutrophils, and activated dendritic cells. In contrast, negative correlations were observed between core genes and the estimated infiltration scores of regulatory T cells, mast cells, and resting dendritic cells. These results suggest that the identified core genes are associated with specific immune infiltration patterns in psoriasis.

### 3.6 Single-cell RNA-seq analysis of the five core genes

**FIGURE 7:**
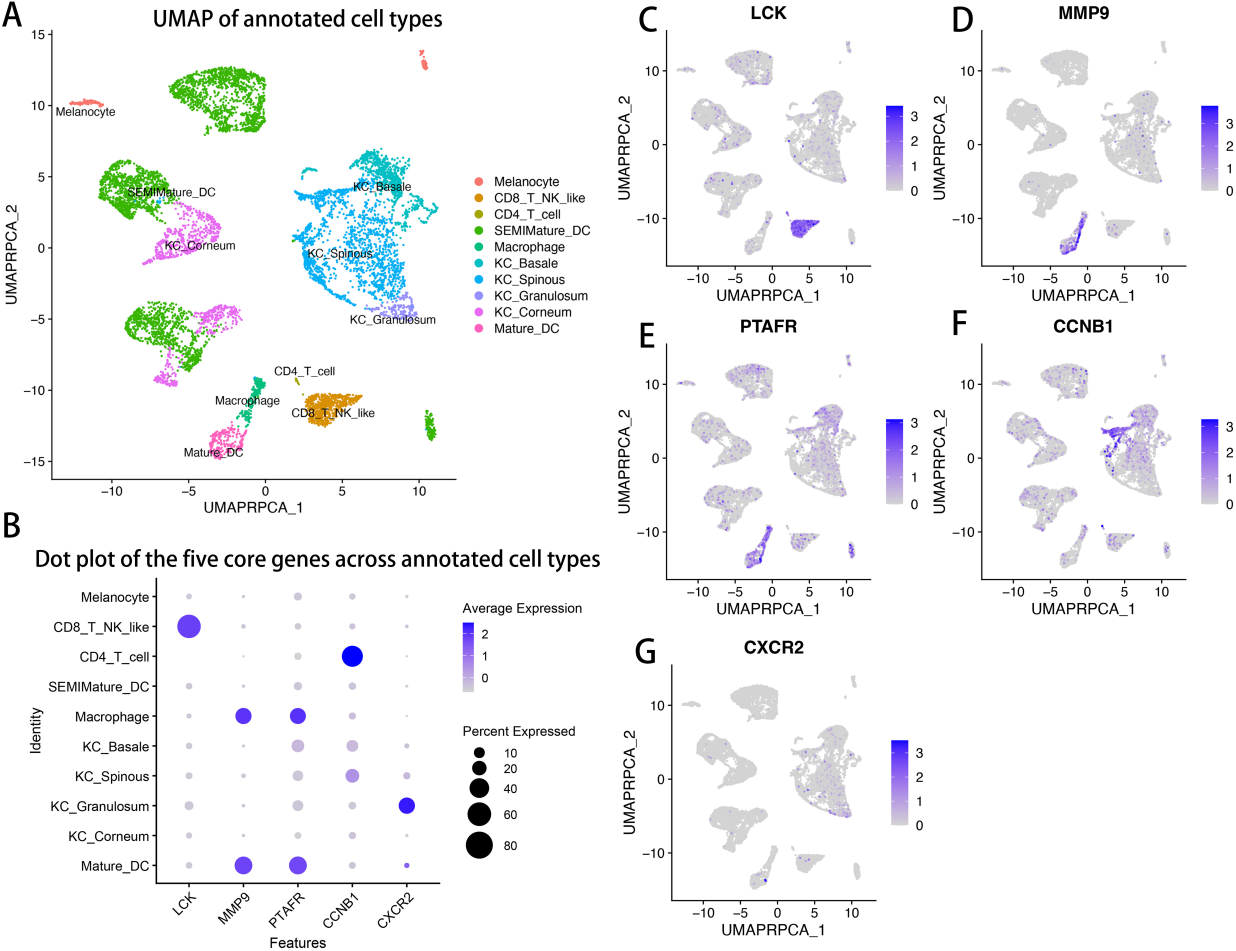
Single-cell RNA-seq analysis of the five core genes in psoriatic tissue. **(A)** UMAP visualization of annotated cell populations identified from psoriatic tissue, including keratinocyte subsets and immune cell populations. **(B)** Dot plot showing the expression distribution of LCK, MMP9, CXCR2, PTAFR, and CCNB1 across annotated cell types. Dot size indicates the percentage of cells expressing each gene, and color intensity indicates average expression level. **(C–G)** Feature plots showing the spatial expression patterns of LCK **(C)**, MMP9 **(D)**, PTAFR **(E)**, CCNB1 **(F)**, and CXCR2 **(G)** across annotated cell populations.

Single-cell transcriptomic data from psoriatic tissue were quality-controlled and processed for downstream clustering analysis. UMAP analysis identified 10 annotated cell populations, including four keratinocyte subsets together with multiple immune cell populations (Figure 7A). Dot plot (Figure 7B) and feature plot (Figures 7C–G) analyses showed that the five core genes displayed distinct expression patterns across keratinocyte and immune compartments. Among them, CXCR2 showed the clearest keratinocyte-associated pattern and was mainly enriched in KC_Granulosum, whereas CCNB1 displayed broader expression across keratinocyte clusters. PTAFR was also detectable in keratinocyte subsets, although its expression was more prominent in macrophage and dendritic cell populations. In contrast, LCK was mainly enriched in CD4_T cells and CD8_T_NK-like cells, and MMP9 was predominantly expressed in macrophages. Overall, these results indicate that the identified core genes are distributed across both epidermal and immune compartments, with CXCR2 and CCNB1 showing relatively closer associations with keratinocyte populations.

### 3.7 Molecular docking analysis of TCDD with identified psoriasis-related targets

**FIGURE 8:**
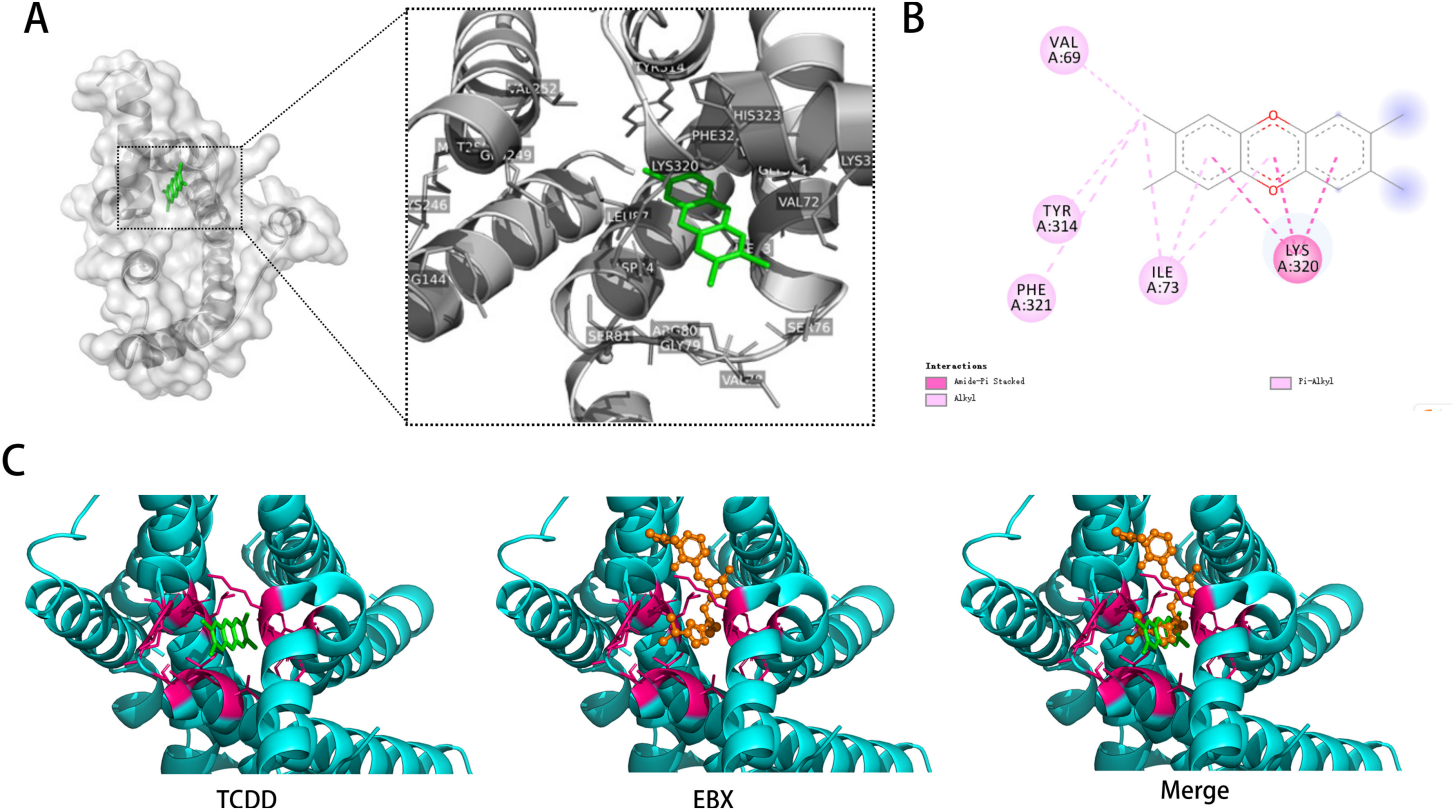
TCDD docks to the EBX-defined intracellular pocket of CXCR2. **(A)** Overview of the CXCR2 structure showing the top-ranked docking pose of TCDD (green) and a magnified view of the binding cavity. **(B)** Two-dimensional interaction map highlighting key pocket residues involved in TCDD binding. **(C)** Structural comparison of the docking pose with the co-crystallized ligand EBX (orange): left, TCDD-bound model; middle, EBX in the 6LFL structure; right, merged view illustrating that TCDD occupies the same intracellular allosteric binding site (IABS) defined by EBX.

Molecular docking was conducted within the network toxicology framework to evaluate the potential interactions between TCDD and four identified psoriasis-related targets (LCK, MMP9, CXCR2, and PTAFR), while CCNB1 was retained as a key regulatory node for network and expression analyses rather than docking, given its primary role as a protein–protein interaction partner lacking a well-defined small-molecule binding pocket (26). TCDD showed favorable predicted docking to all docked targets, with docking scores of −7.8 kcal/mol for CXCR2 (Figures 8A,B), and −7.2, −7.0, and −6.1 kcal/mol for PTAFR, MMP9, and LCK, respectively (Supplementary Figure S3). In general, docking energies below −5.0 kcal/mol are often interpreted as suggesting potential binding feasibility, whereas scores below −7.0 kcal/mol may indicate relatively stronger binding propensity (27).

Among them, CXCR2 exhibited the lowest binding energy (-7.8 kcal/mol), showing the most favorable docking score among the candidates for TCDD. Notably, in the CXCR2 crystal structure (PDB: 6LFL), the top-ranked TCDD pose was predicted to reside within the intracellular allosteric binding site (IABS) defined by the co-crystallized ligand EBX (an IABS antagonist in 6LFL). Superposition analysis indicated substantial overlap between TCDD and EBX within the same cavity and surrounding residues, supporting the plausibility that TCDD may engage the EBX-defined IABS *in silico* (Figure 8C).

### 3.8 Molecular dynamics simulations and binding free energy assessment

**FIGURE 9:**
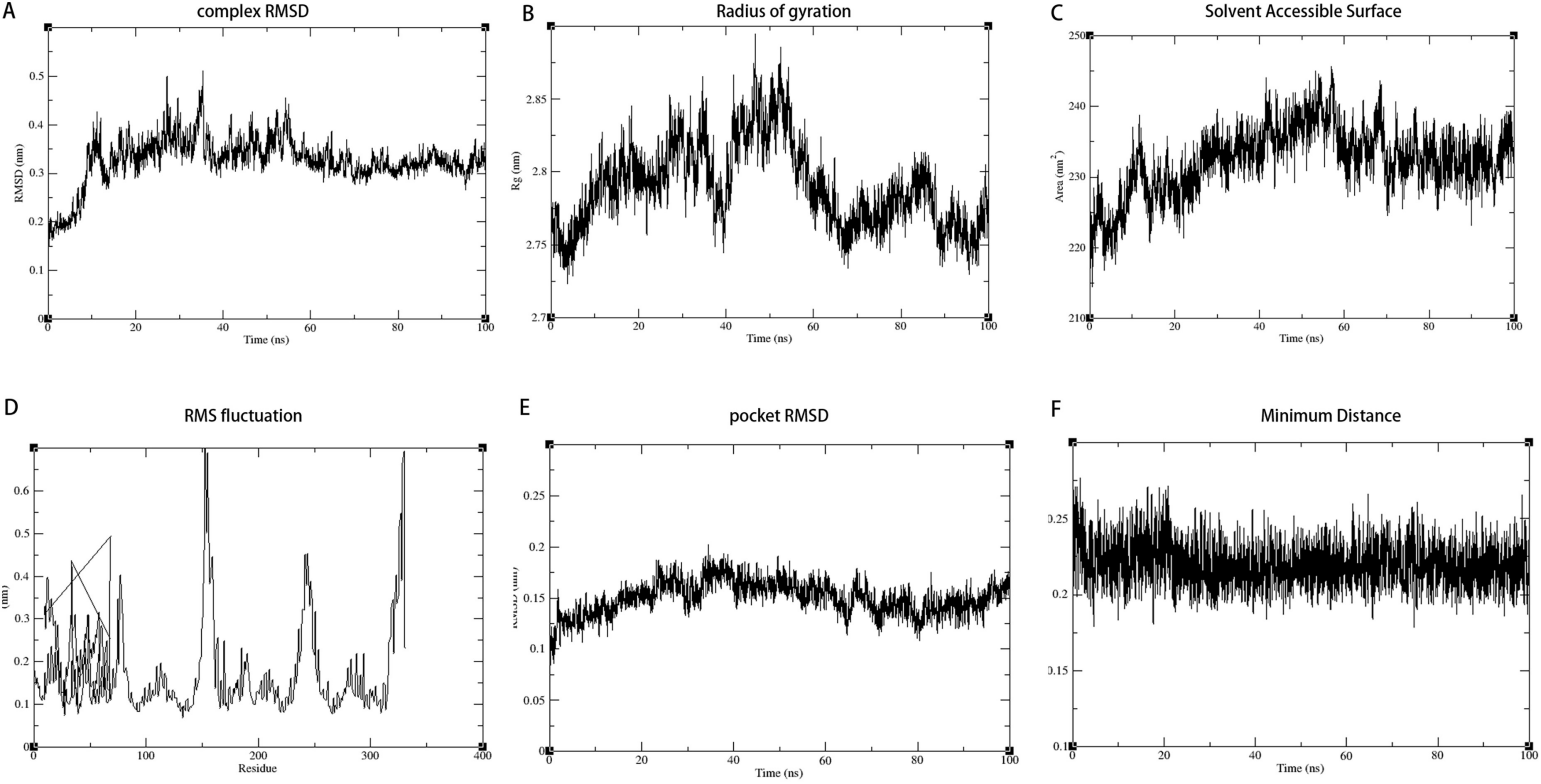
Molecular dynamics simulation of the TCDD–CXCR2 complex. **(A)** RMSD of the complex during the 100-ns simulation. **(B)** Rg of the complex. **(C)** SASA of the complex. **(D)** RMSF of the complex residues. **(E)** RMSD of residues forming the binding pocket. **(F)** Minimum distance between TCDD and CXCR2 during the simulation.

As molecular docking alone cannot fully account for protein flexibility and solvent effects, MD simulations were performed to further assess the stability of the TCDD-CXCR2 complex. During the 100-ns simulation, the RMSD of the complex fluctuated within a narrow range of 0.28–0.35 nm (Figure 9A), indicating a relatively stable protein-ligand system. Consistently, the radius of gyration (Rg) and solvent-accessible surface area (SASA) varied within limited ranges without progressive drift (Figures 9B,C), suggesting no marked global unfolding or expansion during the simulation. RMSF analysis showed generally low residue fluctuations, with higher flexibility mainly observed at terminal and loop regions (Figure 9D), whereas the pocket RMSD remained low (∼0.12–0.18 nm) and the minimum ligand–pocket distance was stably maintained (∼0.20–0.24 nm), supporting a sustained proximity of TCDD to the binding pocket throughout the trajectory (Figures 9E,F). Finally, molecular mechanics/generalized Born surface area (MM/GBSA) provides model-dependent estimates and should be interpreted as supporting relative energetic trends rather than absolute affinities. MM/GBSA calculations showed a favorable binding free energy (ΔGbind = −29.09 kcal/mol), predominantly driven by van der Waals interactions (Table 1), indicating that non-polar contacts may contribute substantially to the predicted association.

**TABLE 1:** Molecular mechanics/generalized Born surface area (MM/GBSA) analysis of the TCDD–CXCR2 complex.

Energy component	Energy (kcal/mol)
ΔEMM (kcal/mol)	−32.71
ΔEEL (kcal/mol)	0
ΔEGB (kcal/mol)	7.65
ΔENPOLAR (kcal/mol)	−4.03
ΔVDWAALS (kcal/mol)	−32.71
ΔGGAS (kcal/mol)	−32.71
ΔGSOLV (kcal/mol)	3.62
ΔTotal (kcal/mol)	−29.09

ΔEMM, molecular mechanical energy; ΔEEL, electrostatic energy; ΔEGB, polar solvation energy (GB); ΔENPOLAR, non-polar solvation energy; ΔVDWAALS, van der Waals energy; ΔGGAS = ΔVDWAALS + ΔEEL; ΔGSOLV = ΔEGB + ΔENPOLAR; ΔTOTAL = ΔGGAS + ΔGSOLV.

### 3.9 Transcriptomic concordance and keratinocyte validation of dioxin-associated responses

**FIGURE 10:**
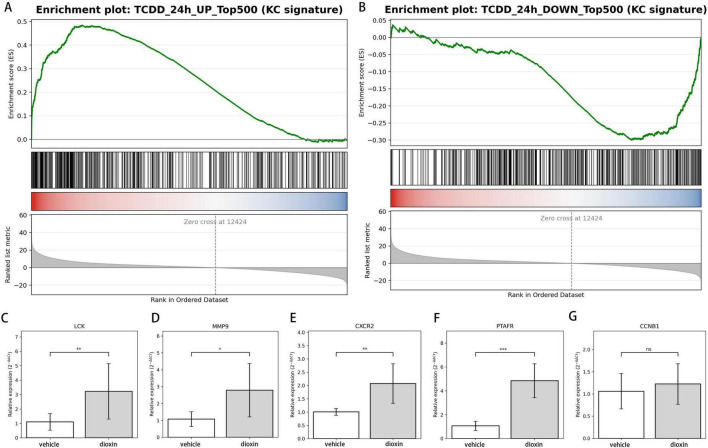
Transcriptomic concordance of keratinocyte TCDD responses with psoriasis and qRT–PCR validation in HaCaT cells. **(A,B)** Preranked GSEA comparing the 24-h keratinocyte TCDD-response signatures (Top500 UP/DOWN from GSE226045) with the psoriatic lesional ranked transcriptome (merged GSE13355 + GSE14905). **(C–G)** qRT–PCR analysis of LCK, MMP9, CXCR2, PTAFR, and CCNB1 in HaCaT cells after 24 h treatment with dioxin or vehicle, normalized to GAPDH (2^-ΔΔCt). Mean ± SD; two-tailed unpaired *t*-test (*P < 0.05, **P < 0.01, ***P < 0.001; ns).

While structural modeling supports the plausibility of TCDD–target engagement, we further sought transcriptomic and experimental evidence for psoriasis-relevant dioxin-associated programs in keratinocytes. Using GSE226045, we derived a 24-h TCDD-response signature by ranking genes in the TCDD versus time-matched control comparison and defining the Top500 upregulated and Top500 downregulated gene sets. Preranked GSEA against the psoriatic lesional ranked transcriptome revealed significant concordance between TCDD responses and psoriasis lesions: the TCDD-24 h UP set was positively enriched toward the top of the psoriasis ranked list (Figure 10A; ES = 0.427, NES = 1.91, FDR = 3.33 × 10^−4^), whereas the TCDD-24 h DOWN set was negatively enriched toward the bottom (Figure 10B; ES = −0.390, | NES| = 1.99, FDR = 3.33 × 10^−4^). Similar directionality was observed when the signatures were expanded to the Top1000 UP/DOWN genes. suggesting that the overall concordance pattern was not driven by the Top500 cutoff. Notably, none of the five core genes were included in the Top500 UP gene set; however, all five ranked within the top 25% of TCDD-responsive transcripts at 24 h, with exact ranks provided in Supplementary Figure S4. These findings suggest that the core genes represent a focused subset embedded within a broader TCDD-induced program.

We next performed *in vitro* validation using dibenzo-p-dioxin, the parent scaffold of TCDD, to assess dioxin-associated modulation of the core genes in keratinocytes. Dibenzo-p-dioxin was selected because of its structural similarity to TCDD, overlapping predicted targets, and comparable docking profiles in our supplementary analyses (Supplementary Figure S5), together with its lower biological toxicity and safer experimental handling. Following 24 h exposure, dibenzo-p-dioxin increased LCK, MMP9, CXCR2, and PTAFR expression compared with time-matched controls, whereas CCNB1 exhibited only a modest change (Figures 10C–G). Together with the transcriptomic concordance analysis, these findings support the plausibility of a psoriasis-relevant dioxin-associated response program in keratinocytes and provide supportive validation for the regulation of several TCDD-prioritized core genes.

### 3.10 Association of TCDD-related core genes with therapeutic response

**FIGURE 11:**
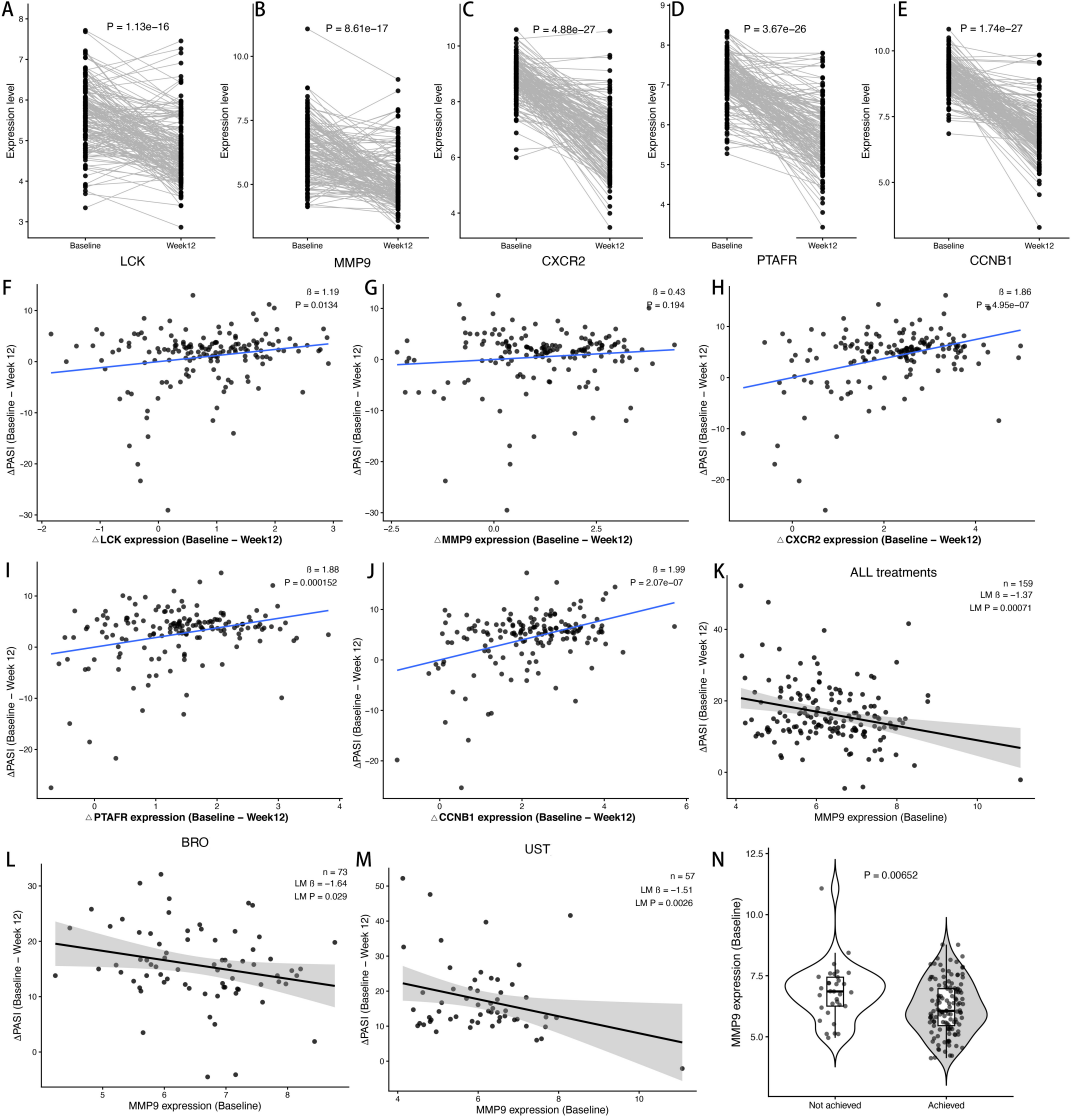
Association of TCDD-related core genes with therapeutic response. **(A–E)** Paired expression levels of LCK, MMP9, CXCR2, PTAFR, and CCNB1 in lesional skin at baseline and week 12. **(F–J)** Associations between treatment-induced transcriptional changes and clinical improvement. **(K)** Association between baseline MMP9 expression and ΔPASI across all treatments. **(L–M)** Treatment-stratified analyses showing the negative association between baseline MMP9 expression and ΔPASI in brodalumab-treated (BRO; L) and ustekinumab-treated (UST; M) patients. **(N)** Violin plots comparing baseline MMP9 expression between PASI75 non-responders (Not achieved) and responders (Achieved).

Having established psoriasis-relevant TCDD responses at the transcriptomic and cellular levels, we next evaluated the clinical relevance of the TCDD-related core genes in biologic-treated psoriasis cohorts. Transcriptomic data from GSE117468 and GSE117239 were integrated after batch-effect correction to investigate the involvement of TCDD-related core genes in therapeutic response (Supplementary Figure S2). In this pooled cohort of patients treated with cytokine-targeted biologics, paired analyses of lesional skin samples demonstrated significant downregulation of all five core genes after 12 weeks of therapy (Figures 11A–E). Next, we assessed whether treatment-induced transcriptional changes were associated with clinical improvement. PASI improvement was positively correlated with the extent of post-treatment decreases in LCK, CXCR2, PTAFR, and CCNB1 (Figures 11F,H–J), suggesting that effective suppression of these genes was linked to greater clinical benefit. In contrast, changes in MMP9 expression were not significantly correlated with PASI improvement (Figure 11G).

We then examined whether baseline expression levels of the five core genes were associated with therapeutic response. Notably, higher baseline MMP9 expression was significantly associated with smaller PASI improvement at week 12 (FDR = 0.0035; Figure 11K), whereas no significant associations were observed for the other core genes. This negative association was consistently observed in both brodalumab- and ustekinumab-treated patients (Figures 11L,M). Consistently, patients achieving PASI75 response exhibited significantly lower baseline MMP9 expression than non-responders (Figure 11N). ROC analysis further suggested a modest discriminatory ability of baseline MMP9 expression in identifying PASI75 responders (AUC = 0.65; Supplementary Figure S6). Collectively, these findings indicate that baseline MMP9 was associated with week-12 clinical improvement and may represent a candidate marker associated with treatment response in biologic-treated psoriasis cohorts, although its discriminatory performance for PASI75 response was modest.

## 4 Discussion

Dibenzo-p-dioxins, particularly TCDD, have been implicated in cutaneous toxicity, inflammatory signaling, and immune dysregulation (12), yet the downstream molecular programs through which these environmentally responsive signals may relate to psoriasis remain insufficiently defined. In the present study, we used an integrated systems-level strategy to examine how TCDD-associated targets and pathways may intersect with psoriasis-related molecular networks. Our findings suggest that TCDD-associated signals may converge on inflammatory and epidermal programs relevant to psoriasis, thereby providing a framework for understanding how environmental toxicant exposure could be linked to disease-related immune dysregulation and treatment response.

Using network toxicology, we identified 87 candidate genes potentially associated with TCDD-related psoriasis. Functional enrichment analyses indicated that these genes were mainly involved in IL- 17-, chemokine-, and MAPK/ERK-related signaling, suggesting convergence on inflammatory and immune-regulatory programs relevant to psoriasis. These pathway-level findings support the view that TCDD may be linked to psoriasis-related immune dysregulation through coordinated effects on chemotactic and pro-inflammatory signaling rather than through a single pathway (28). Within the canonical TCDD–AhR framework, this pattern is biologically plausible, as AhR acts as an upstream xenobiotic sensor that links environmental toxicant exposure to epidermal, inflammatory, and immune transcriptional programs (13). In this context, the enrichment of xenobiotic-response, chemokine-, IL- 17-, and MAPK/ERK-related pathways in our overlapping gene set is compatible with a model in which TCDD–AhR signaling contributes to psoriasis-relevant epithelial and inflammatory responses.

Using LASSO and SVM-RFE, we identified five core genes—LCK, MMP9, CXCR2, PTAFR, and CCNB1—that were persistently highly expressed in psoriatic skin. The strong diagnostic performance of these genes suggests their potential relevance as candidate biomarkers in the analyzed datasets; however, because feature selection and diagnostic evaluation were both performed within the same merged cohort, independent external validation will be necessary to confirm their robustness and generalizability. Importantly, these core target genes were closely associated with the characteristic immune cell infiltration patterns of psoriatic inflammation. Their expression levels were positively correlated with pro-inflammatory immune cell populations, while negatively correlated with regulatory or resting immune cell subsets. These associations suggest that the identified gene signature may be linked to inflammatory immune infiltration patterns in psoriasis. Mechanistically, the five genes map well onto key elements of psoriatic inflammation: LCK is linked to T-cell activation and Th17-related responses (29), CXCR2 and MMP9 align with neutrophil recruitment and tissue remodeling (30, 31), PTAFR connects lipid-mediator signaling to inflammatory cell activation, and CCNB1 is consistent with the hyperproliferative keratinocyte program (32, 33). However, because the immune analysis was based on deconvolution-derived infiltration estimates and correlation analysis, these findings should be interpreted as associations rather than direct evidence of functional immune reprograming.

At the cellular level, single-cell analysis further refines the biological interpretation of the core-gene signature by showing that these genes are distributed across both keratinocyte and immune cell populations in psoriatic tissue, rather than being restricted to a single cellular compartment. This pattern suggests that the identified core genes reside at the epithelial–immune interface that characterizes psoriatic lesions and supports the view that TCDD-associated molecular programs may converge on keratinocyte–immune crosstalk. However, because the present single-cell analysis was based on psoriatic tissue rather than direct TCDD perturbation at single-cell resolution, these findings should be interpreted as providing cellular context for the identified targets.

Subsequent molecular docking analysis showed that TCDD can adopt energetically favorable binding poses across core targets (docking scores < −5 kcal/mol), with CXCR2 displaying the most favorable docking score among the candidates. Notably, structural mapping using the CXCR2 crystal structure (PDB: 6LFL) showed that the top-ranked TCDD pose occupied the intracellular allosteric binding pocket (IABS; a cytoplasmic small-molecule modulatory site) defined by the co-crystallized ligand EBX, with substantial spatial overlap within the same cavity (34). This observation is mechanistically relevant because the IABS can modulate GPCR conformational states and downstream coupling. We therefore conducted MD simulations and MM/GBSA analyses to test whether this docking pose remains stable in a dynamic, solvated environment. MD simulations supported a persistent TCDD–CXCR2 interaction at the EBX-defined IABS, with stable pocket RMSD and consistently low ligand–pocket minimum distance, indicating no dissociation over 100 ns. MM/GBSA suggested a favorable binding free energy dominated by van der Waals contributions, consistent with a non-polar binding mode. These results support the plausible hypothesis that TCDD may interact with CXCR2 in a manner that could influence receptor dynamics relevant to chemokine-driven inflammation.

We further strengthened the biological plausibility of this network by integrating transcriptomic concordance analysis and keratinocyte validation. The concordance between the keratinocyte TCDD-response program and the psoriatic lesional transcriptome suggests that TCDD exposure can initiate a disease-relevant molecular state in keratinocytes, a key epithelial compartment in which xenobiotic sensing can be translated into inflammatory outputs. In line with our enrichment results highlighting xenobiotic response and downstream transduction modules (e.g., MAPK/ERK) together with immune/chemotactic signaling, the enrichment of TCDD-upregulated genes among psoriasis-upregulated transcripts indicates that TCDD tends to shift keratinocytes toward a lesion-like transcriptional direction. Such epithelial reprograming could prime keratinocytes to both produce and respond to inflammatory mediators, thereby facilitating a keratinocyte–immune cell feed-forward loop that amplifies chemotactic trafficking and tissue inflammation. In parallel, keratinocyte validation using dibenzo-p-dioxin, the parent scaffold of TCDD, showed increased expression of LCK, MMP9, CXCR2, and PTAFR, whereas CCNB1 exhibited only a modest change. Together, these findings support the plausibility of a psoriasis-relevant dioxin-associated response program in keratinocytes and provide supportive validation for the regulation of several TCDD-prioritized core genes. Accordingly, future work should extend these observations to patient-derived lesional tissues and single-cell analyses to better define the cellular context and translational relevance of TCDD-associated responses in psoriasis.

We further explored whether TCDD-related genes influence clinical treatment response in psoriasis. Notably, effective biologic therapy was accompanied by a coordinated suppression of the TCDD-associated core gene module, tracking with clinical improvement as reflected by PASI reduction. This pattern suggests that pollutant-linked immune activation is not merely a fixed imprint of exposure history, but is dynamically modulated by the inflammatory tissue milieu that is reshaped during therapeutic resolution. Among the identified core genes, higher baseline expression of MMP9 was consistently associated with diminished clinical improvement. Mechanistically, MMP9 is a well-established effector output of chemokine-driven neutrophil activation (35). In psoriasis, neutrophil-derived MMP9 has been implicated in vascular/endothelial dysfunction and inflammatory amplification, and MMP9 levels correlate with disease activity and decrease with effective anti-inflammatory therapy (36). Prior work further shows that TCDD can directly enhance MMP9 expression and activity (32), supporting a mechanistic bridge between pollutant sensing and matrix-remodeling inflammatory effector pathways. Collectively, these findings suggest that MMP9 may serve as a candidate indicator of pollutant-associated inflammatory burden and may have potential relevance as a stratification biomarker for treatment responsiveness, pending further validation.

Several limitations of this study should be acknowledged. First, this study relied on retrospective analyses of publicly available transcriptomic datasets and individual-level TCDD exposure data were unavailable; therefore, exposure–response relationships could not be established. Second, the experimental validation was restricted to HaCaT keratinocytes, limiting the translational relevance of the findings; future validation of TCDD-related responses in patient-derived lesional samples is warranted. Finally, therapeutic-response analyses involved heterogeneous biologic regimens and limited covariates; residual confounding cannot be excluded, and the discriminatory performance of baseline MMP9 was modest, its predictive utility should be validated in independent cohorts with harmonized treatment and standardized sampling.

In conclusion, our findings support a plausible link between TCDD-associated targets and psoriasis-related immune dysregulation. The identified core genes and pathways may be involved in inflammatory and keratinocyte-associated responses relevant to psoriasis pathogenesis and therapeutic response. These observations remain hypothesis-generating and warrant further mechanistic and clinical validation.

## Data Availability

The data presented in the study are deposited in the Gene Expression Omnibus (GEO) repository (https://www.ncbi.nlm.nih.gov/geo/), accession numbers GSE13355, GSE14905, GSE117468, GSE117239, GSE162183, and GSE226045. The original contributions presented in the study are included in the article/Supplementary material. Further inquiries can be directed to the corresponding author.
