# Supplementary material for: Integrated systems toxicology identifies TCDD-responsive targets linked to immune dysregulation and treatment response in psoriasis
Source: Front Med (Lausanne). 2026 May 29;13:1816748. doi: 10.3389/fmed.2026.1816748 (PMC13259674; doi:10.3389/fmed.2026.1816748)

Supplementary Material

# Supplementary Data: List of the 87 overlapping genes identified as TCDD-associated targets in psoriasis.

EGFR, CA2, ESR1, MMP1, HMGCR, NR3C1, ERBB2, CYP1A2, LCK, FYN, ADRB2, ELANE, CTSG, PTPRC, CYP3A4, ADORA3, CYP2C9, DRD2, MMP9, PRKCA, FLT1, CNR1, ACHE, PTGS1, CXCR1, CXCR2, EDNRA, TACR1, PTAFR, MAPK3, HTR2A, HTR2C, MAPK1, CASP1, BDKRB2, SLC6A4, MC4R, CYP2C19, PTGS2, HRH1, OPRM1, AHR, OPRK1, HTR2B, CCR2, CCR4, CCR5, KCNH2, MAPK14, ESR2, CYSLTR1, CYP1A1, ARNT, BRCA1, CYP1B1, HSP90AA1, AHRR, CDK1, CDK7, CCNB1, CTSS, CTSL, CTSB, BACE1, CTSC, KAT5, TGFBR1, TRPA1, MMP2, MIF, PPARG, PDE4A, MMP13, MMP3, CCR1, KDR, ABCC8, KCNJ11, ADAM17, S1PR1, ICAM1, SELE, MAPK8, CA9, ERN1, HTR1A, PDE7A.

Supplementary Table 1. Basic information of datasets used in the study.

| Datasets | Sample size | | | | Platform |
| --- | --- | --- | --- | --- | --- |
|  | Normal | | Psoriasis | |  |
| GSE13355 | 64 | | 58 | | GPL570 |
| GSE14905 | 21 | | 33 | | GPL570 |
| GSE162183 | 3 | | 3 | | GPL24676 |
|  | | | | | |
|  | Treatment (week 12) | | | |  |
|  | Ustekinumab | brodalumab | | Etanercept |  |
| GSE117468 | 15 | 73 | | / | GPL570 |
| GSE117239 | 42 | / | | 29 | GPL570 |
|  | | | | | |
|  | DMSO | | TCDD | |  |
| GSE226045 | 2 | | 2 | | GPL18573 |

Supplementary Table 2. Rank positions of the five core genes in the 24-h TCDD-response transcriptome.

| **Gene** | **rank** | **Top percent** |
| --- | --- | --- |
| **CXCR2** | **757** | **Top 1.30%** |
| **CCNB1** | **1145** | **Top 1.96%** |
| **PTAFR** | **4947** | **Top 8.49%** |
| **MMP9** | **6450** | **Top 11.07%** |
| **LCK** | **12539** | **Top 21.51%** |

# Supplementary Figures

Supplementary Figure S1. KEGG pathway enrichment dot plot of overlapping TCDD–psoriasis genes.

**
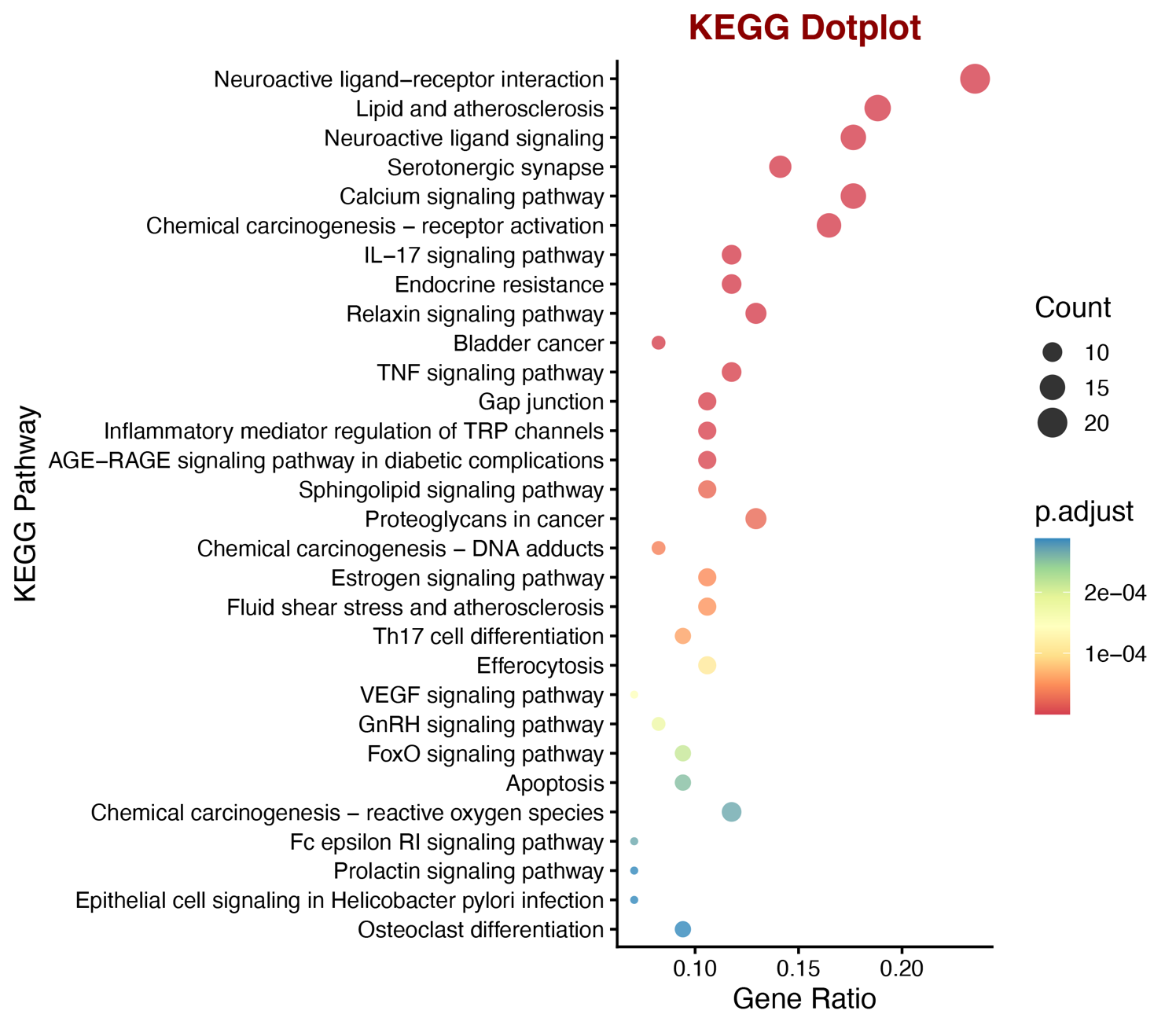
**

Supplementary Figure S2. Assessment of batch effects before and after batch correction in psoriasis transcriptome datasets.
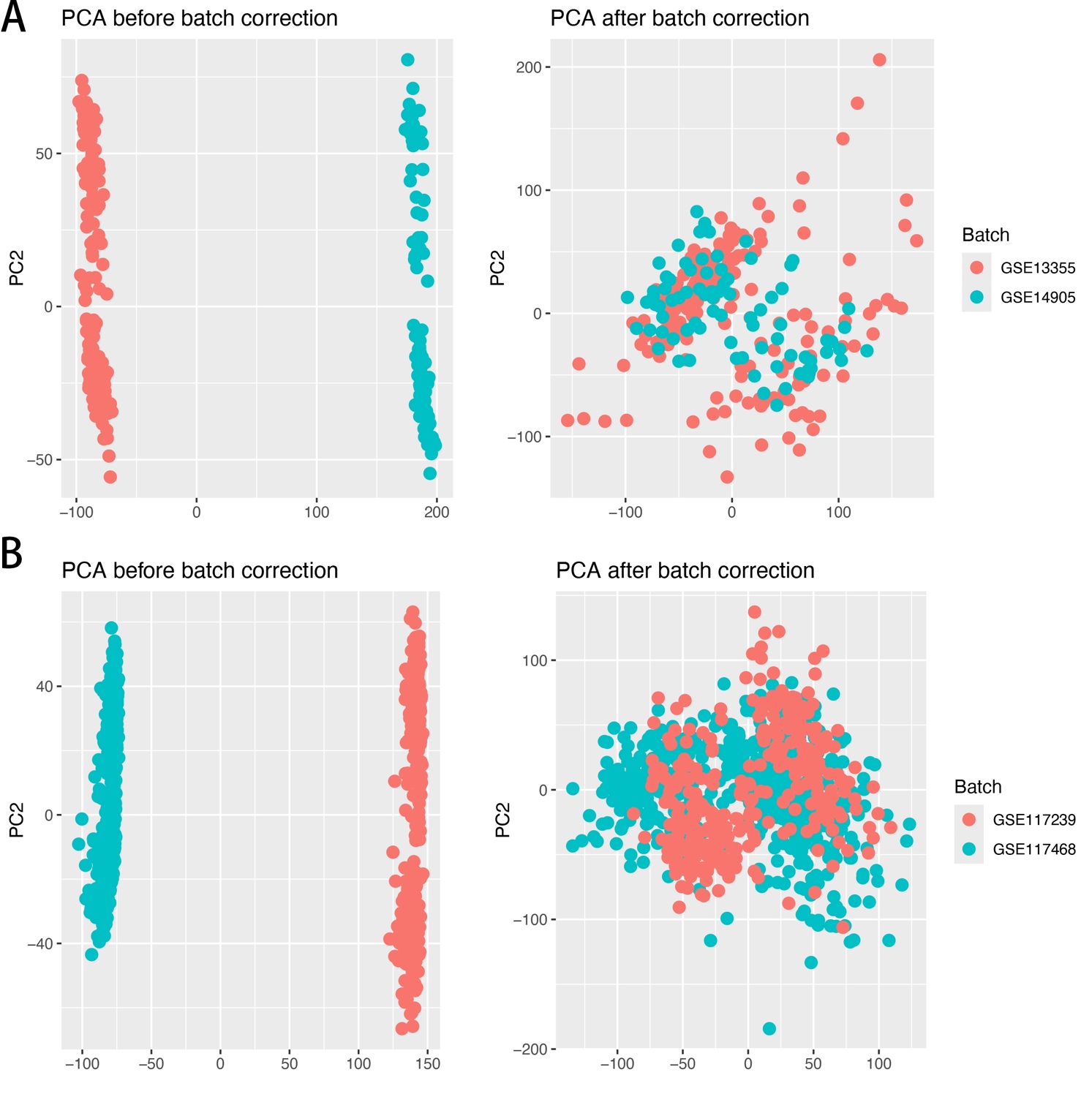


Supplementary Figure S3. Molecular docking analysis showing the docking poses of TCDD with core target proteins: (A) PTAFR, (B) MMP9, and (C) LCK.
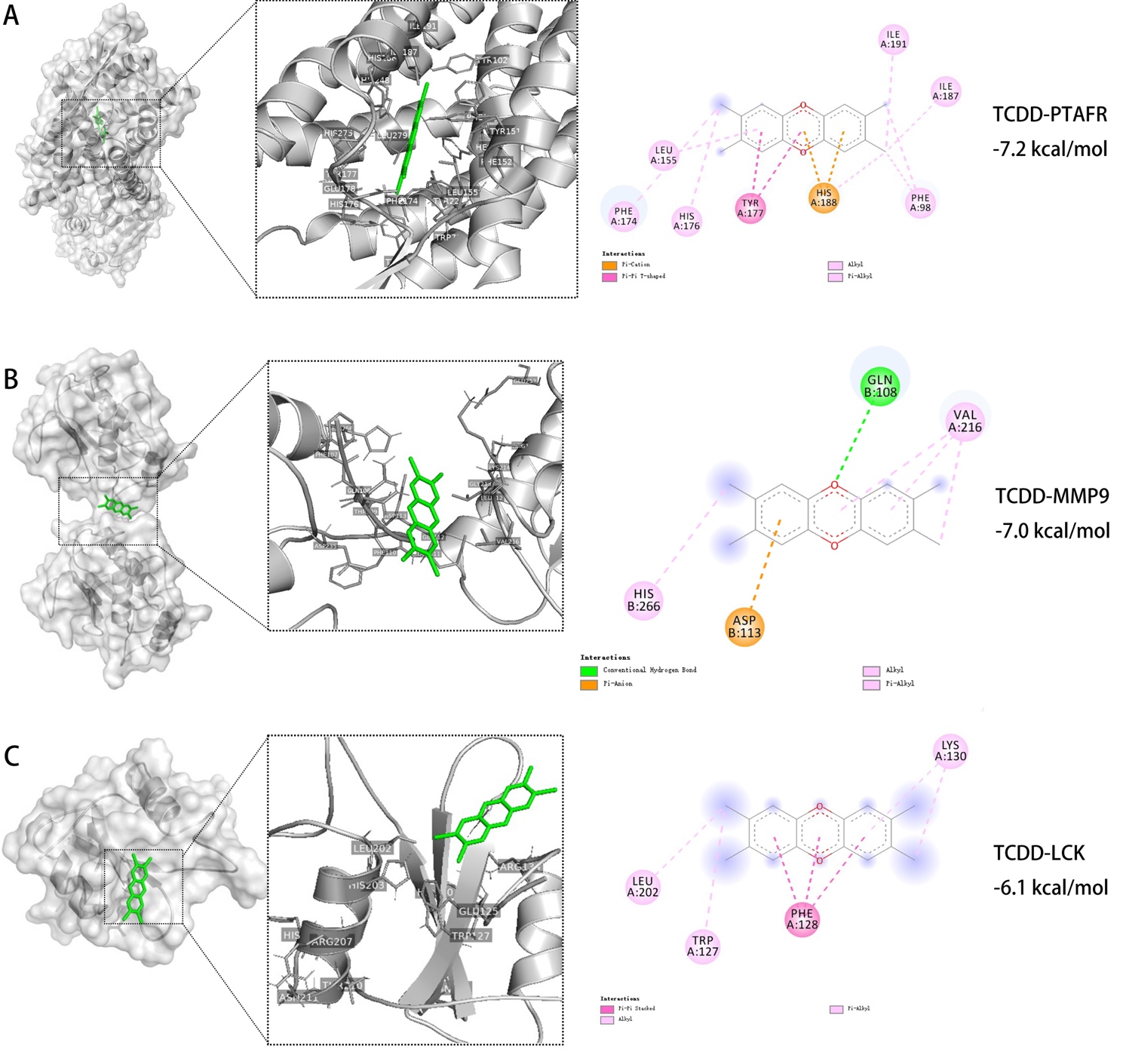


Supplementary Figure S4. Preranked GSEA comparing the Top1000 TCDD-response signatures (UP and DOWN) from GSE226045 with the psoriatic lesional ranked transcriptome.


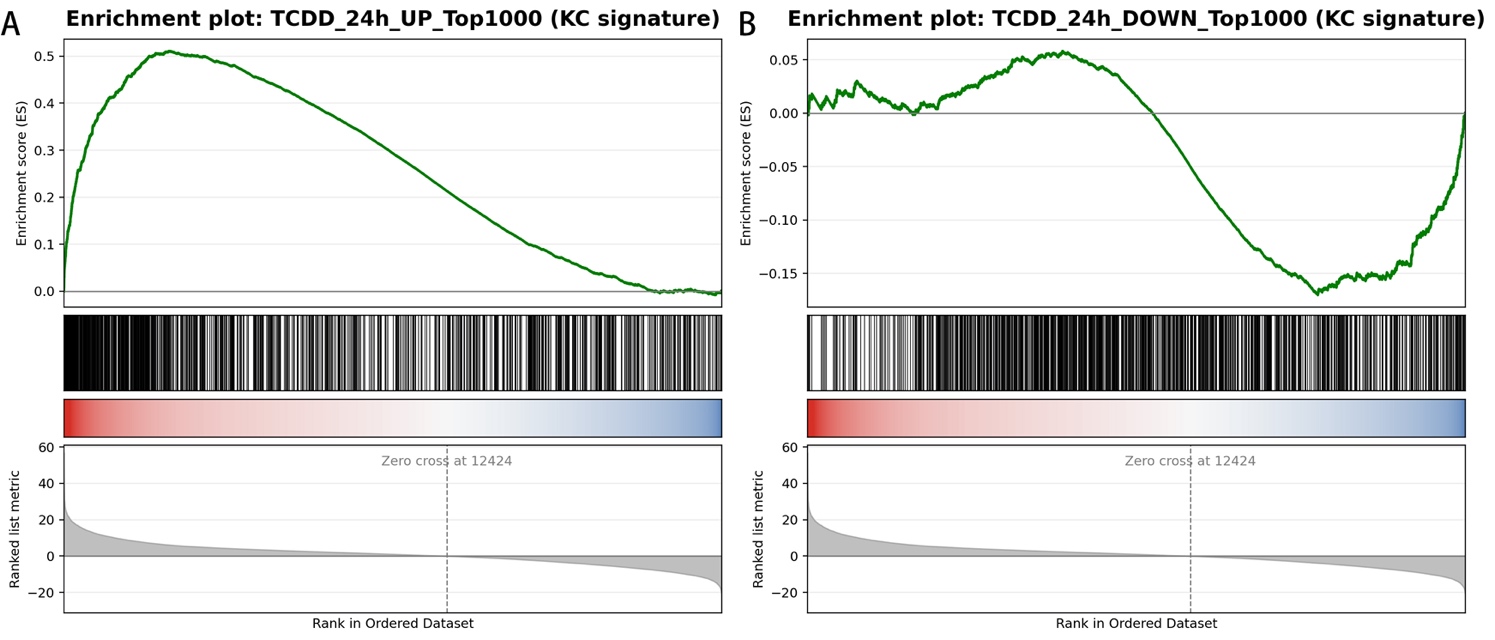


(A–B) Preranked GSEA comparing the expanded 24-h keratinocyte TCDD-response signatures (Top1000 UP and Top1000 DOWN from GSE226045) with the psoriatic lesional ranked transcriptome (merged GSE13355 + GSE14905). The Top1000 UP signature showed positive enrichment toward the top of the psoriasis ranked list (A), whereas the Top1000 DOWN signature showed negative enrichment toward the bottom (B), supporting that the overall concordance pattern was not driven by the Top500 cutoff.

Supplementary Figure S5. Structural and in silico comparison between dibenzo-p-dioxin and TCDD.


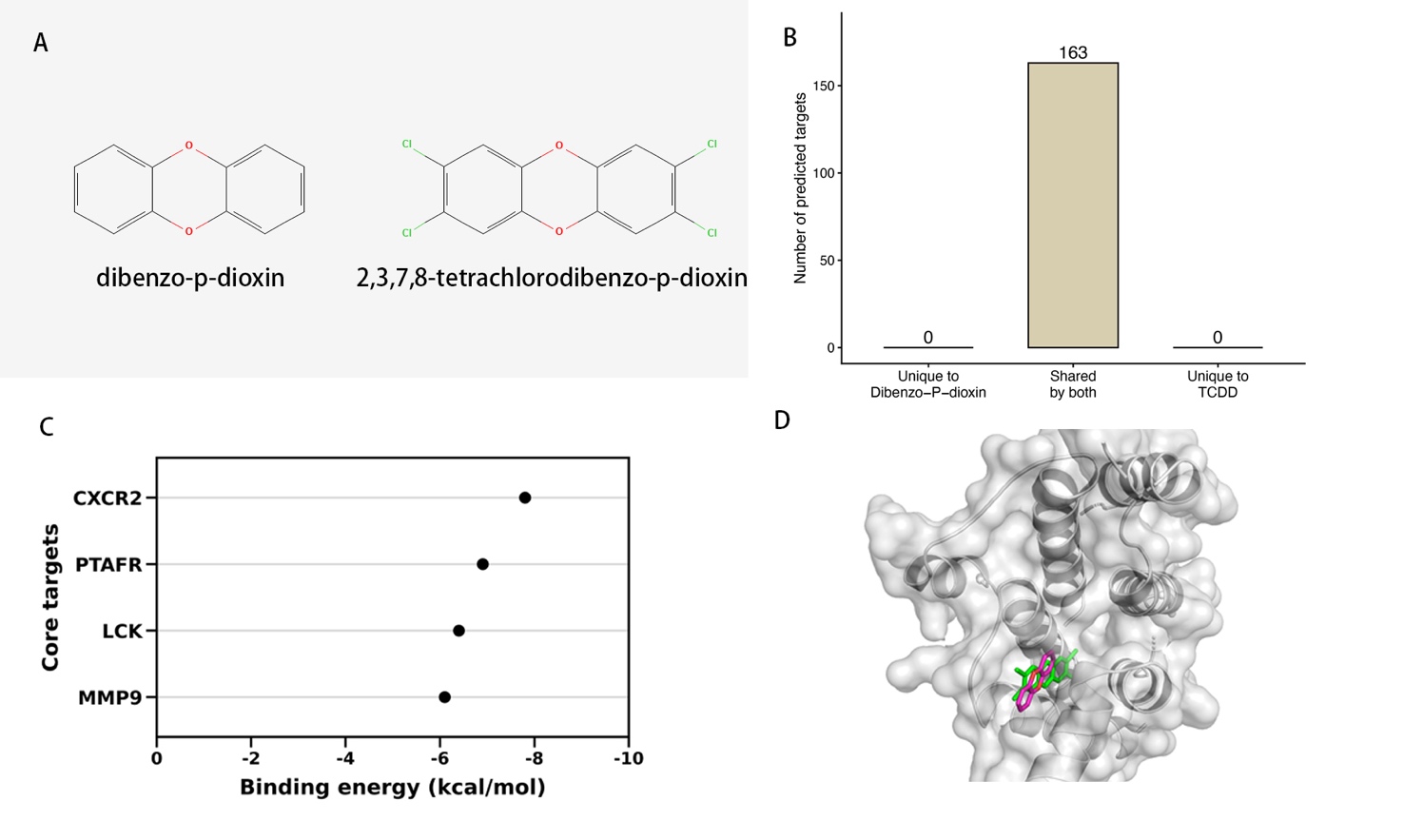


(A) Chemical structures of dibenzo-p-dioxin and 2,3,7,8-tetrachlorodibenzo-p-dioxin.

(B) Comparison of predicted targets showing complete overlap between dibenzo-p-dioxin and TCDD.

(C) Predicted docking energies of dibenzo-p-dioxin with the core targets.

(D) Superposition of dibenzo-p-dioxin and TCDD docking poses in CXCR2 showing similar binding positions.

Supplementary Figure S6. ROC curve evaluating the ability of baseline MMP9 expression to discriminate PASI75 responders at week 12.


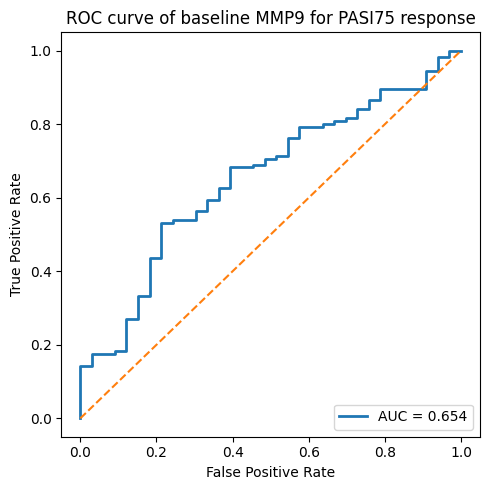

Supplement: Supplementary file 1 [file Data_Sheet_1.docx]
